# Observations on Phlegmatia Dolens

**Published:** 1801-09

**Authors:** 


### *Observations on Phlegmatia Dolens, by* Dr. Hull.

[ Continued from our laſt, pp. 135—151. ]

Mr. WHITE, in the former part of his *Inquiry, &c.* has never alluded to a diviſion of the lymphatics, which deſcend into the cavity of the pelvis, in conſequence of a ſharp line or ridge of the os pubis; but thinks it probable that "the *predis-*

*ponent*

*ponent caufe* may be a weaknefs of the coats of the lymphatics in fuch fubjects only as have thefe veffels formed into one principal trunk under Poupart's ligament," p. 55; and he intimates, that the remote caufe of the difeafe under confideration, is a rupture of one or more lymphatic veffels, "owing to the child's head preffing the lymphatic veffel or veffels, which arife from one of the lower extremities againft the brim of the pelvis during a labour pain, fo as to ftop the progrefs of the lymph," p. 49.

In my Effay on Phlegmatia *dolens*, I have offered fome objections to the above account of the production of the difeafe, and to other parts of the Theory, as given by Mr. White in his former publication. Thefe objections he has endeavoured to obviate, in the Second Part of his *Inquiry*, *&c.* p. 78, &c. and we fhall now fee with what fuccefs.

My *firft* objection is, that "*there is no evidence of the burfting of a lymphatic during labour, much lefs of its healing in fuch a manner as to render the canal impervious, or occafion a diminution of its diameter.*" Mr. White replies, "There is the evidence of Mr. Aftley Cooper, that the contractile power of the abforbents is fufficient to occafion a rupture of their coats by tying them." Now, Mr. Cooper's Experiments, which are referred to by Mr. White in proof of this affertion, only fhew this, viz. that the *receptaculum chyli* of a dog, which is large and thin in this animal, is generally, but not always, found ruptured when the thoracic duct is tied. They do not fhew that *the lymphatic of the extremities* have been ruptured, in confequence of a ligature paffed round them, or the thoracic duct; nor has Mr. White adduced a fingle experiment to prove that this has ever been the cafe. There is no part of the lymphatic fyftem of the lower extremities of a woman, that bears the leaft analogy in its ftructure to the *receptaculum chyli* of a dog; confequently, what Mr. White has here adduced, is totally irrelevant. Mr. A. Cooper obferves, that at the lower part of the neck, the thoracic duct is generally divided into two or three trunks, which again become united into one; and he thinks it probable, that this may ferve as a provifion againft difeafe, from what happened to him in tying the thoracic duct of a dog; for having included only one of thofe divifions, the two others carried the chyle into the blood, and the animal recovered.* I have lately been informed by Mr. A. Cooper, that the legs of the dog, which was the fubject of the above experiment, did not become fwelled.

The

* Medical Records and Refearches, p. 101 and 102.

The ſame ingenious anatomiſt tied the extremity of the thoracic duct near the left jugular and ſubclavian veins in another dog: The duct became much diſtended, and the dog died forty-eight hours after. On diſſection, the receptaculum chyli was found ruptured, and an effuſion of chyle had taken place. The abſorbent veſſels of the hinder extremities and organs of generation were diſtended, but not in an equal degree with thoſe of the left fore leg and left ſide of the neck; one of the latter was larger than a crow's quill.* None of the legs of this dog were materially ſwelled, as he ſince informed me.

My ſecond objection is, that "*there is not the leaſt reaſon to ſuppoſe, that a lymphatic trunk ever burſts in conſequence of the tranſient obſtruction of the lymph, induced by a labour pain. If this temporary obſtruction, which does not occaſion any ſenſible enlargement of the limb could produce the rupture of a lymphatic, we might expect, from the long continuance of the obſtruction, which takes place agreeably to Mr. White's hypotheſis, that every lymphatic in the limb would burſt, before the limb had arrived at half the ſize which we know it attains in the diſeaſe under conſideration.*" Mr. White replies in the following words: "Mr. A. Cooper has likewiſe proved, (See note, p. 64) that the tranſient obſtruction of the lymph, for a few minutes, will burſt an abſorbent. Whenever the ſwelling of the limb loſes its elaſticity, and pits upon preſſure, it is probable that many of the lymphatic veſſels are burſt." This reply does not obviate, or even bear upon my objection. Mr. A. Cooper's experiment, to which Mr. White alludes, only proves, that by the compreſſion of the thoracic duct for a few minutes, the *receptaculum chyli* of a dog may be found ruptured.†

My *third* objection is, "*If the ſwelling of the limb be occaſioned by a congeſtion and coagulation of lymph in the abſorbents, how is it poſſible it could ever ſubſide? How is it poſſible, that the ſwelling ſhould be ſo nearly even as it is found to be at firſt? And why can we not trace the gradual increaſe of the lymphatics in every inſtance, from a ſlightly enlarged or varicoſe ſtate, to that extreme degree of diſtention at which they muſt arrive, in order to contain all the fluid neceſſary for the production of this enormous ſwelling of the limb.*" Mr. White, in anſwer to the firſt query, ſays, "The abſorbents are certainly as capable of abſorbing coagulated lymph as coagulated blood, but they are equal to ten times more than that." I am ready to grant, that the abſorbents, *when their canals are pervious*, are capable

* Medical Records and Reſearches, p. 107.

† Ibid, p. 110.

ble of abforbing not only coagulated blood, but flefh and bone; neverthelefs, when thefe are completely gorged with coagulated lymph, as Mr. White fuppofes them to be, I do not fee how they are to be freed from it. To the fecond query he replies, "The fwelling being fo nearly even, is owing to the fuperficial lymphatic veffels in the extremities of the human body being fo very numerous, fourteen trunks frequently accompanying one vein:" But from the obfervations of Soemmering, and other acute anatomifts, we find, that the lymphatics, when much diftended, do really become varicofe and perceptible in limbs not affected with Phlegmatia *dolens*. Why then cannot they be conftantly perceived in a varicofe ftate in limbs affected with this difeafe? If the lymphatics, as Mr. White fuppofes, contain all the additional matter of the limb in cafes of Phlegmatia *dolens*, they muft, I conceive, become as much diftended as varicofe veins are, which, we know, become extremely enlarged, without adding much to the thicknefs of the inferior extremities. To the third query he anfwers, "he can trace the gradual increafe of the lymphatics, in every inftance, from the groin to the ends of the toes." The increafe of the limb from above, downwads, may be traced; but I much doubt, whether in any inftance of this difeafe, the lymphatics have been traced, gradually increafing in this direction; for I cannot admit, that the mere enlargement of the limb proves the gradual increafe of the lymphatics; much lefs can I allow, that the lymphatics are ever fo far diftended as to contain the quantity of matter depofited and accumulated in the limb. I am of opinion, if the lymphatics of the limb of a dog, or any other animal, be tied, and a confiderable intumefcence of the limb take place, in confequence of the interruption given to the courfe of the lymph, that the cellular membrane of the leg will be found much loaded with the exhaled fluid.

My *fourth* objection is, "*It does not appear, that in any of the fourteen cafes particularly related by Mr. White, or in any of the fourteen communicated to him, an enlargement of the lymphatic veffels was at any period obferved.*" This has not been farther noticed by Mr. White than in his anfwer to the foregoing objection.

From what has been alleged, I am led to conclude, 1ft. that confiderable obftruction, and evident diftention of the lymphatic veffels do frequently occur, without producing Phlegmatia *dolens*; 2dly. that this difeafe very frequently occurs without any perceptible enlargement of the lymphatic veffels; and, 3dly. that if the lymphatics, in certain cafes of Phlegmatia *dolens*, have been, or fhall be found increafed in fize, this is rather rather to be confidered as an accidental, than as an effential circumftance, or as a confequence rather than as a caufe of the difeafe.

### III. *Mr. White's Objections to the Theory of Phlegmatia Dolens, given in my Essay, considered.*

As my Theory is given very fully, and with its illuftration, occupies no lefs than eleven pages, I muft beg leave to refer the reader to my Effay, and fhall only ftate in this place, that I confider the proximate caufe of the difeafe, as confifting in *an inflammatory affection, producing fuddenly a confiderable effufion of ferum and coagulating lymph from the exhalents into the cellular membrane of the parts affected.* To this theory Mr. White has raifed various objections, in the Second Part of his *Inquiry*, p. 32, &c. which I fhall now proceed to examine.

He begins with faying, "There can be no inflammation in any part of a human or animal body, wherein red blood is ufually circulated, but it will fhew itfelf by an increafed colour of the part, either of a red, purple, or bluifh tint. Red blood is not ufually circulated in the cornea, the pleura, nor the peritonæum; but when thefe parts are inflamed, a rednefs frequently takes place, in confequence of fome red blood in them. There can be no inflammation in the external parts of mufcles, cellular membrane, or any part of the fkin; in the fuperficial blood veffels, cutaneous lymphatics, in their fuperficial trunks, or in the glands of the leg and thigh; but it may, in every inftance where it occurs, be feen in every living human fubject." Mr. White may properly be afked here, what kind of affection takes place in the limb of a perfon afflicted with acute rheumatifm, when there is pain, tendernefs, rigidity, tumour, and heat, without any red, blue, or purplifh tint to be feen? or, what is the affection of a conglobate gland, which occurs in confequence of a chancre, and is attended with pain, heat, and enlargement, without any difcolouration of the fuperincumbent fkin? It is to be remembered, that the cutis is not tranfparent, and confequently cannot indicate the difcolouration of the fubjacent inflamed parts, except when the inflammation has extended to its upper furface. Though no external rednefs appears in the two cafes mentioned above, there is, I conceive, an inflammatory affection of the mufcles and ligament, &c. in the former cafe, and of a lymphatic in the latter cafe; and, I have no doubt, but this would be fhewn by diffection. Neither have I any doubt, that a limb, affected with Phlegmatia *dolens*, if diffected in the beginning of the difeafe, would exhibit unequivocal vocal marks of inflammation. I think I have accounted fatisfactorily for the outer furface of the fkin not being reddened in this complaint.* That Mr. W. has expreffly admitted the prefence of inflammation in Ph. *dolens*, will now be fhown from his own words. In the former part of his *Inquiry*, *&c.* where he is treating of the cure of this malady, he very properly obferves, that it muft vary according to the different ftages; and adds, "The firft, which may be called the *inflammatory*, muft be treated in the antiphlogiftic method; but as this *inflammation* is not the original difeafe, but a *fymptom* only, occafioned by the diftenfion of the lymphatic veffels and glands, it is not neceffary or prudent to wafte the patient's ftrength by large evacuations," p. 59. The point then, properly at iffue betwixt Mr. White and me, is not, whether there be an inflammatory affection of the limb and other parts, but whether the inflammation be the primary complaint, or only a fymptom arifing from diftenfion? To determine this point, let us firft advert to what takes place in the lower extremities, when affected with anafarca, and afterwards with Phlogofis *erythema*. In this cafe the anafarca is very properly confidered as protopathic, and P. *erythema* as deuteropathic; becaufe the pain, heat, and rednefs, &c. characterizing the latter difeafe, are fubfequent to the fymptoms which mark the prefence of anafarca, and evidently originate from the irritation produced by the diftenfion of the fkin. On the contrary, in Phlegmatia *dolens*, the pain heat, tendernefs, &c. precede the intumefcence, and are relieved by it; confequently, the inflammatory affection muft be the primary one, and not a fymptom only, as Mr. W. has here affumed. When Ph. *erythema* occurs as a fymptomatic affection, it is neceffary that it be marked by the fame concourfe or affemblage of fymptoms, as when it occurs as a primary affection, and the fame may be faid of other fymptomatic affections: Therefore, if inflammation occur as a fymptom of Phlegmatia *dolens*, it muft be marked by the fame concourfe of fymptoms which conftitute inflammation, when occurring as an original difeafe. Now Mr. White has actually ftated, that there is inflammation in Phlegmatia *dolens*; and as he has alfo ftated, that the fkin is whiter than natural in this difeafe, he muft have formed an idea of inflammation which does *not* "fhew itfelf by an increafed colour of the part, either of a red, purple, or bluifh tint;" and admitting the exiftence of inflammation in this difeafe, he could not well give it a feat different from what I have done in my Effay.

* See Effay on Phlegmatia Dolens, p. 209, &c.

Pyrexia *preceding* or accompanying the complaint, "continues Mr. White," is not an unerring fymptom of inflammation, either acute or chronic, by fome called paffive, efpecially when there is no hard, ftrong, full pulfe, no rednefs, itching, nor throbbing of the part, no plethoric ftate of the general fyftem, nor any partial plethora, which are none of them *common attendants* upon this difeafe. "It would appear then from this conceffion alfo, that fymptoms of inflammation are *occasional*, if not *common* attendants on this diforder.

"The excruciating pains, ftiffnefs, tendernefs, increafed heat, and fwelling of the parts may," according to Mr. White, "be accounted for on other principles." I anfwer, not fatisfactorily; for unlefs he can make it appear that effects precede their caufes, his own explanation muft be rejected.

The ingenious author next chufes to employ the interrogatory mode of attack. He puts no fewer than twelve queftions in immediate fucceffion, all of which I fhall now confider, in the order he has placed them.

*Question* 1. "If the *lymph* coagulate in the cellular membrane, why does this happen in the beginning, rather than towards the termination of the diforder?" *Answer.* By *lymph*, properly fpeaking, is underftood a fluid that is, or has been, contained in the lymphatics or abforbents. Now I have no where faid, that the *lymph*, taken in this fenfe, does coagulate in the cellular membrane of a limb affected with Ph. *dolens*. I maintain, that in addition to the exhaling fluid, ufually poured into the cellular membrane, a quantity of *coagulating lymph*, or *fibrine*, according to the new chemical nomenclature, is thrown from the exhalants into the cellular membrane of the affected parts, and there coagulates in the beginning of this difeafe; but that afterwards, only the common exhaling fluid, or even a more watery humour, is thrown into the cellular membrane. If Mr. White wifhes to know, why fibrine is effufed in the beginning rather than towards the termination of the diforder, I reply in his own words, that in the firft ftage there is inflammation, and that in the third or laft ftage, "pain and fever have entirely left the patient, and no complaint remains except the fwelling of the limb, and perhaps a *general relaxation*." *Inquiry*, &c. Part I. p. 64. Every perfon acquainted with the practice of phyfic knows, that when the membranes of cavities are inflamed, fibrine or coagulating lymph is thrown into thefe cavities by the exhalants, as in Peritonitis *puerperalis*, Phlegmatia *dolens*, &c. and that a thin watery fluid is poured into them by the exhalants, as in afcites, anafarca, &c. when thefe veffels are in a relaxed ftate.

*Question*

*Question* 2. "If the lymph is contained in the cells of the cellular membrane, if we obtain one drop by puncture, why is the quantity limited?" *Answer.* As the fibrine and other fluids, effused into the cellular membrane, coagulate; if a puncture be made through the skin in the first stage of the complaint, only a small quantity of serum, or serosity, issues from it in general: But the quantity of watery fluid which is thus discharged in a more advanced stage of the disease, is greater, because the coagulated and fluid parts are then more distinct, and the proportion of liquid to the coagulum is also increased, owing to the effusion of fibrine ceasing, whilst that of the exhaled fluid continues, or is increased. It is probable that adhesion takes place in consequence of the inflammation, and that the communication betwixt the different parts of the cellular membrane is thence rendered less free than natural.

*Question* 3. "Why is its absorption not promoted by the common means." I have yet to learn that it is not. I think it very probable, that the absorbents take up and transmit more exhaled fluid throughout the whole course of this disease, to the thoracic duct, than in the natural state of the parts affected, and that the intumescence is produced and kept up by a materially increased effusion from the inflamed exhalants in the first stage, and from the relaxed exhalants in the last stage of this complaint.

*Question* 4. "If the coagulating lymph be thrown out in consequence of a greater determination of blood, why does it not take place more early?" *Answer.* Mr. White's cases shew, that the disease takes place as early as in twenty-four hours after delivery;* that it also occurs at the end of the first, second, third, and fourth week, and as late as five weeks †. This is just what we should expect, from considering the disease as an inflammatory affection, depending upon a greater determination of blood to the parts, and occasioning an effusion of coagulating lymph, or fibrine, into the cellular membrane. If the exciting causes be early applied, the disease will appear early; if late, the disease will appear late. For further particulars relative to the remote causes of the disease, I must beg leave to refer to my Essay.

*Question* 5. "Why does not the same occurrence sometimes take place after tapping for the dropsy of the ovarium, amputation, or the operation for the popliteal aneurism?" *Answer.* I

* See the case of Betty Grime. *Inquiry*, Part 1. p. 33.

dare not ſay, that the diſeaſe does not ſometimes take place after theſe operations. If Ph. *dolens* were capable of being produced by injuries of the lymphatics, it ſhould frequently ſucceed to the operation for the popliteal aneuriſm, becauſe the deep ſeated lymphatics which accompany the femoral artery muſt frequently, if not always, be divided or tied in this operation. Why the diſeaſe occurs moſt frequently in lying-in women, I have endeavoured to explain in my Eſſay.

*Queſtion* 6. Why does it ever happen to women during pregnancy, when an oppoſite ſtate muſt prevail?" *Anſwer.* What Mr. White means by an *oppoſite ſtate*, I do not, perhaps, exactly comprehend He ſurely will not contend, that pregnant women are exempt from inflammatory complaints.—He here indirectly admits, that the diſeaſe does occur during pregnancy; an admiſſion, which at once ſhews the impropriety of the name he has given it, and the improbability of his theory, as far a relates to the production of Phlegmatia dolens. That the diſeaſe does take place during pregnancy is alſo proved by the caſes related by Dr. Evans and Puzos; and I have lately been made acquainted with a caſe, the ſubject of which was a lady, who was neither pregnant nor lying-in, nor had received any external injury, at leaſt this laſt circumſtance is not intimated to have happened. The caſe of Mr. R. related by Mr. White, appears to have been a caſe of this diſeaſe, and I underſtand from Mr. White, that Mr. Eaton, who attended this gentleman with him, conſiders it as ſuch. Now, if this were actually a caſe of Phlegmatia dolens, it muſt be admitted as proving that the diſeaſe is not excluſively confined to the female ſex, and as ſhewing further the impropriety of naming it *Phlegmatia alba dolens puerperarum.* That there was a fracture of the pelvis is not improbable from Mr. White's account of the caſe; but as he did not aſcertain the ſituation of the fracture, it cannot, without further evidence, be admitted, that the fracture was ſituated where Mr. White ſuppoſes it to have been from the view of a ſkeleton, much leſs that the lymphatic veſſels, which deſcend into the tubular part of the pelvis, were ruptured or lacerated by the fractured os pubis. He aſks, "How can we poſſibly account for the ſwelling of *the left ſide of the ſcrotum only* at the expiration of a fortnight, but from the rupture of the lymphatic veſſels of that ſide of the ſcrotum, and the mouths of thoſe veſſels healing in that time and *totally* obſtructing the paſſage of the lymph?" Another perſon may aſk, how can it be accounted for in this way, ſince the ſuperficial lymphatics of one ſide of the ſcrotum anaſtomoſe with thoſe of the other ſide, and ſince the ſuperficial lymphatics of one ſide anaſtomoſe with the deeper ſeated lymphatics of the ſame ſide, and theſe laſt paſs with the lymphatics of the teſticle into the abdomen and not under Poupart's ligament? Beſides Mr. W. has declared, that there was not "any ſwelling or inflammation in the glands," p. 121, and he makes no mention of having traced or obſerved any enlargement of the abſorbents of the ſcrotum; yet, according to his theory of the diſeaſe, both the inguinal glands and the lymphatic trunks ſhould have been enlarged in this caſe. It is remarkable that Mr. White makes the following poſitive aſſertion relative to the caſe of Mr. R. after ſaying that nothing can be *more ſimilar* than this caſe is to the *Phlegmatia alba dolens Puerperarum*: "Here was the ſame kind of ſwelling confined to the inguinal, hypogaſtric, and lumbar regions, and the whole limb and genitals on one ſide. The lymphatic veſſels *were ruptured in the ſame place and by the ſharp edges of the ſame bone*, with this difference only, that in one it is ſharp by nature, in the other it was become ſo by being fractured," p. 123. And afterwards, conſcious, as it would ſeem, that he was ignorant whether this were the caſe or not, he ſays, "*If it be granted me that this ſwelling was occaſioned by the broken bone* injuring the lymphatics, how can we account for it upon any other principle than that the lymphatic veſſels, which paſs over and wrap round the body of the os pubis, were ruptured by the ſharp edges of that broken bone, the mouths of which were healed up and rendered impervious?" p. 124.—He afterwards ſays, "If it had proceeded from inflammation only, it would ſcarcely have continued ſo long as ſeveral weeks," p. 125.—For my own part, I cannot perceive that any argument worth attention can be drawn from the continuance of this ſwelling for ſeveral weeks againſt its having proceeded from inflammation. And upon what ground will Mr. W. deny that this affection of Mr. R's limb, &c. was an inflammatory affection beginning in the injured parts, extending down the limb, and accompanied by an effuſion of fibrine and other fluids into the cellular membrane? Mr. White has ſtated, in the quotation given above, that the ſwelling was confined to the inguinal, hypogaſtric and lumbar regions, and *the whole limb and genitals on one ſide.*—A queſtion may therefore ariſe, whether one half of the penis was affected in this caſe? he informs us that neither of the teſticles were. Another queſtion may alſo ariſe, reſpecting the parts affected in women labouring under Phlegmatia *dolens*, viz. whether this diſeaſe affected only one of the nymphæ and one ſide of the clitoris, meatus urinarius, and vagina, in thoſe caſes wherein Mr. White found only one labium vulvæ affected?

After this digreſſion I ſhall now return to the remaining queſtions propoſed by Mr. White.

*Queſtion* 7. "Why does it generally only take place in one limb, limb, or in the other when the firſt is recovering?" *Anſwer.* In inflammatory diſeaſes it very frequently happens that only one ſide or part of the body is affected; thus, for example, in Peripneumonia *Pleuritis*, one ſide of the thorax only is affected; and we very frequently find one teſticle, one eye, one ear, one kidney, one breaſt, &c. only inflamed. If Mr. White can account for this, he will alſo be able to account for one limb being affected in Phlegmatia dolens. If the lymphatics be divided on both ſides during labour, why does not the diſeaſe occur in both ſides at the ſame time?

*Queſtion* 8. "Why the lymphatics of one ſide only ſhould be filled is evident; but why the blood-veſſels of one ſide ſhould have the inflammation confined to them, when the ſame cauſes have been applied to both, and they communicate with each other, is not ſo obvious?" I wiſh to know what reaſon Mr. White has for ſaying, that *the ſame cauſes* have been applied to the blood-veſſels on both ſides, when Phlegmatia *dolens* only takes place in one? There muſt, I conceive, be a difference with reſpect to the cauſes, or the ſame effects would follow in both limbs, &c. That the blood-veſſels communicate by means of anaſtomoſes is true, and we have Mr. White's authority for ſaying that the lymphatics "are known to be *more numerous* and to anaſtomoſe *more frequently* than the arteries, p. 73.—He agrees even with Mr. Cruikſhank, that the ſuperficial lymphatics in the extremities of the human body are very numerous, "fourteen trunks frequently accompanying one vein." *Inquiry*, *&c.* p. 8 . Hence it will appear from Mr. White's own words, that the lymphatics communicate more frequently with each other than either the arteries or veins. How then is it *ſo eaſy* to conceive that the lymphatics of one ſide only may be affected, and *ſo difficult* to conceive that the blood veſſels of one ſide only ſhould be affected? Again, does not inflammation frequently attack one inferior extremity without affecting the other? Does it not ſeize the leg without affecting the thigh of the ſame ſide? Does it not frequently affect the lower part of the leg without extending to the upper part of the ſame leg? If Mr. White can account for theſe limited inflammatory affections, I preſume he will be able to account for Phlegmatia *dolens* being, in general, more or leſs confined to one ſide, yet ſometimes extending to both.

*Queſtion* 9. "If it proceeds from a general cauſe, why are its limits ſo certainly defined? *Anſwer.* The limits of the diſeaſe, as I have already proved, are not ſo exactly and certainly defined as Mr. White has been pleaſed to ſtate. It may not be improper to adduce here Levret's account of the manner in which the diſeaſe paſſes from one ſide to the other.—"Mais ſi, des dès le commencement de la diminution de la cuisse, les sueurs ne se declarent pas, & que les urines, ou les selles ne deviennent pas plus abondantes & laiteuses, il faut s'attendre que l'humeur ne fait que se déplacer, & qu'elle se déposera bientôt sur quelqu' autre partie. En effet, sa marche la plus ordinaire, en pareil cas, est de passer de la cuisse à la fesse du même côté; elle gagne ensuite le dedans du bassin, puis la fesse & la cuisse du côté opposé & de-là se communique à la jambe & au pied; ensorte que ces différentes parties éprouvent successivement les mêmes symptômes qu'on avoit remarqués dans la premiere extrémité." *L'Art des Accouch.* § §. 941 & 942. How perfectly this case may be explained by considering it as a gradual extension of the inflammatory affection from one limb to the other! How unsatisfactory must every attempt to account for it be, agreeably to Mr. White's hypothesis!

*Question* 10. "Why does it attack the lumbar region, where the determination of blood before delivery would be greater than after? *Answer.* I must beg leave to refer Mr. White to my Essay, p. 202, and p. 264, &c. for the reason why Phlegmatia *dolens* frequently attacks the lumbar region; and I cannot allow, that the determination of blood to the lumbar region is greater before delivery than after. By lumbar region, I mean here those parts of the loins that are affected in Phlegmatia *dolens*, not including those abdominal viscera, which may be said to be situated during pregnancy in the lumbar region.

*Question* 11. "Why does it commence at the upper part, rather than at all points alike?" *Answer.* There is, I believe, no part of the lower extremity, in which this disease may not, or has not begun, and this corresponds perfectly with what ought to be expected from an inflammatory affection. Why it most frequently begins in the lower part of the trunk, I think I have assigned satisfactory reasons in my Essay. If a swelling invade one of the lower extremities from mere obstruction of the lymphatics at the brim of the pelvis, I should expect it to begin sometimes at least at the foot, and to proceed gradually up the limb; to be of the œdematose kind, but rather more firm than œdematose affections generally are when occurring in debilitated habits, and to be attended with little pain, and that pain succeeding to, not preceding the distension of the skin. In short, I should expect the intumescence to resemble Phlegmatia *diuturna* more in its symptoms than Phlegmatia *dolens*.

*Question* 12. "If this disorder be occasioned by a general or local plethora, or there exists a general or local inflammation; why does it ever happen to tender delicate women, exhausted and debilitated by violent floodings and other evacuations? *Answer.* There is, perhaps, no state of the system, however however great the debility, in which inflammation may not take place. We find lying-in women, much weakened by floodings and other evacuations, not uncommonly attacked with Peritonitis *puerperalis*. And it has been ſhewn above, that Mr. White himſelf ſays, there is inflammation in this diſeaſe, and that the firſt ſtage may be named inflammatory, and that he propoſes the antiphlogiſtic mode of treating it. The puerperal ſtate, which is characterized by debility and irritability, and a ſudden change in the determination of the circulating fluids, appears to give a peculiar prediſpoſition to Phlegmatia *dolens*, and to occaſion the inflammatory affection of the lower extremity, &c. to be attended with an extenſive effuſion of fibrine and other fluids into the cellular texture, and to exhibit that concourſe of ſymptoms which conſtitutes the character of the diſeaſe under conſideration.

In page 133 of my Eſſay on Phlegmatia *dolens*, I have ſaid, " It has in many inſtances attacked women who were recovering from puerperal fever, and in ſome caſes has ſupervened, or ſucceeded to thoracic inflammation." Mr. White, in commenting upon this paſſage, makes the following declaration: " That anaſarca, puerperal fever, thoracic inflammation, and *Phlegmatia alba dolens puerperarum* may ſucceed each other, or even exiſt together, I have no reaſon to doubt, as they are by no means incompatible; but as I have never ſeen ſuch occurrences, I ſhould therefore conclude, they are ſo rarely met with, as not to be ſufficient to build a theory upon." *Inquiry*, p. 38.

I have ſufficiently proved the truth of my obſervation by caſes which have occurred to myſelf and others, and I ſhall beg leave to adduce here, two extracts from Mr. White's Inquiry, &c. p. 1, and then leave the reader to determine how far the above aſſertion is ſupported or contradicted by his own caſes. Speaking of Martha Wilkinſon, (Caſe vii. p. 22,) he ſays: " On the ninth day after delivery, ſhe was ſeized with a *pleuritic stitch* on her right ſide; on the thirteenth ſhe was attacked with pain in her left groin, and labium pudendi, &c." Speaking of Elizabeth Rothwell, (Caſe viii. p. 23 and 24,) he ſays: " She was kept very warm, never went out of her room, nor did any thing, by which ſhe could poſſibly catch cold, till twenty days after her delivery, when *a window was opened in the room* about a minute or two. *In an hour after she was seized with a pain in her right side* and ſhoulder, but had no cough. *She was bled, which eased the pain.* Two days after, ſhe was attacked with pain in the groin, labium pudendi, &c."

To conclude, although I have laboured ſtrenuouſly to ſhew, 1ſt, That the affection of the labium pudendi is improperly conſidered

ſidered as the pathognomonic ſymptom of Phlegmatia *dolens* by Mr. White; 2. That my caſes which, he has endeavoured to reject, were true and genuine caſes of the diſeaſe; 3. That his theory is purely hypothetical; and, 4. That the objections which he has raiſed to my theory, have little weight. I hope I have no where departed from that reſpect which is due to a gentleman of Mr. White's age, experience, rank, and abilities, as a writer and practitioner, and who may juſtly be conſidered as having deſerved well of his profeſſion and his country for more than half a century.

